# Decoding C‑SH2 Domain/Peptide Interactions in SH2 Domain-Containing Tyrosine Phosphatase 2: A Molecular Framework for Rational Inhibitor Design

**DOI:** 10.1021/acsomega.5c09452

**Published:** 2026-01-15

**Authors:** Chiara Innamorati, Layla Bruno, Paolo Calligari, Gianfranco Bocchinfuso, Lorenzo Stella

**Affiliations:** Department of Chemical Sciences and Technologies, 9318 University of Rome Tor Vergata, Rome 00133, Italy

## Abstract

SH2 domain-containing tyrosine phosphatase 2 (SHP2), encoded by *PTPN11*, plays a crucial role in multiple cellular processes, including proliferation and differentiation. Mutations in *PTPN11* are implicated in various developmental disorders and hematological diseases, while wild-type (WT) SHP2 is a pivotal target in cancer therapy. SHP2 comprises two Src-homology 2 domains (N-SH2 and C-SH2), followed by a protein tyrosine phosphatase (PTP) catalytic domain. Under basal conditions, the N-SH2 domain autoinhibits SHP2 by blocking access to the catalytic site. An allosteric transition controls the detachment of the N-SH2 domain from the active site (and thus catalytic activity) and the affinity of the N-SH2 domain for its binding partners. We recently introduced the inhibition of protein–protein interactions (PPIs) of SHP2 as a novel, promising pharmacological strategy, an alternative to active site or allosteric inhibition. While our past efforts have focused on targeting the N-SH2 domain, this strategy shows limited efficacy against WT SHP2, where the autoinhibited conformation prevails, and the N-SH2 domain binding site is mostly unavailable. Conversely, the C-SH2 domain is not allosterically regulated, and its binding site is always accessible in both the active and inactive states of SHP2. Targeting this domain represents an alternative strategy to block SHP2 PPIs, allowing inhibition of the WT protein and weakly activated mutants. In this study, by performing molecular dynamics (MD) simulations of selected C-SH2/peptide complexes and by critically analyzing the available data from peptide libraries, the sequences of high-affinity ligands, both natural and artificial, and the experimental structures, we defined the features governing the C-SH2 binding affinity and specificity. Our analysis reveals that residues at positions +1 and +3, relative to the pY, provide hydrophobic stabilization, while polar residues are suitable at +2. The presence of a cationic residue at position +4 allows a gain in selectivity for the C-SH2 domain with respect to N-SH2. Finally, a cationic or aromatic residue at position +5 may contribute to binding affinity and selectivity. Notably, our MD simulations reveal transient but relevant interactions involving N-terminal residues that are not detectable in crystallographic structures. These findings lay the groundwork for designing peptide inhibitors that specifically target the C-SH2 domain of SHP2.

## Graphical abstract

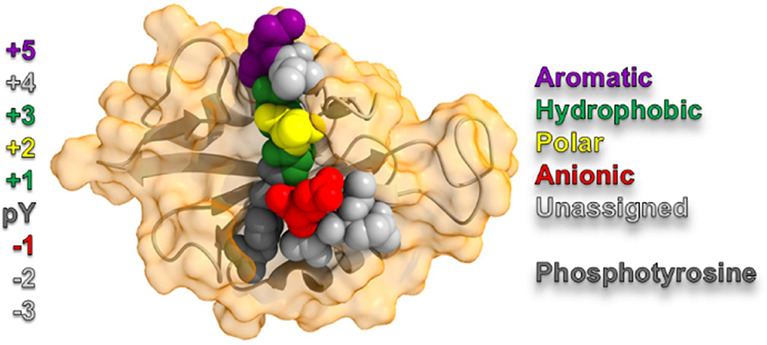

## Introduction

### SH2 Domains as Drug Targets

The discovery of Src-homology 2 domains (SH2) by Pawson’s group in 1986 ^1^ led to the idea of protein modularity, with independently folding domains of conserved sequences.^2^ The name of this family comes from the identification of a conserved sequence of about 100 amino acids in the Src oncoprotein. The “2” suffix indicates that this module is the second in the Src sequence.^3^ Today, it is known that the human genome encodes 121 SH2 domains across 111 different proteins.^4,5^ They are present in adaptors, scaffolds, kinases, phosphatases, proteins involved in signal regulation, transcription, chromatin remodeling, phospholipid second messenger signaling, and cytoskeletal regulation.^6^ Their primary role is to recognize and bind specifically to pY residues in proteins along with adjacent amino acids that define specificity. Furthermore, SH2 domains enhance tyrosine phosphorylation *in vivo* by protecting binding sites in their target proteins from dephosphorylation.^7^ Tyrosine phosphorylation contributes only ∼0.5% of the total phosphoproteome, yet it plays a critical roles in the regulation of eukaryotic cells.^8^ For these reasons, SH2 domains are considered very promising drug targets, particularly in the inhibition of PPIs.^4,9–11^ Mutations affecting the binding properties of SH2 domains are directly involved in several genetic diseases.^12^ In addition, selective SH2 domain ligands would be invaluable tools to study the role of specific PPIs in signal transduction pathways.^13^ However, clinical and research applications of SH2 binders have been limited, particularly because these domains usually display low binding affinity and selectivity.^14–17^

### SH2 Domain-Containing Protein Tyrosine Phosphatase 2 as a Therapeutic Target for Cancer and Rare Diseases

The SH2-containing protein tyrosine phosphatase 2 (SHP2), which comprises two SH2 domains, was the first protein tyrosine phosphatase (PTP) whose gain-of-function mutations were identified as oncogenic.^18^ Generally, protein tyrosine phosphatases are involved in the negative regulation of cell signaling. However, SHP2 is one of the few tyrosine phosphatases that play a positive regulatory role in signal transmission. This protein is ubiquitously expressed and mediates signal transduction downstream of various receptor tyrosine kinases (RTKs), is required for the full activation of the RAS/MAPK pathway,^19^ and modulates signal transduction in other cascades, such as PI3K-AKT and JAK-STAT. Therefore, SHP2 is involved in regulating multiple cell processes, including proliferation, survival, differentiation, and migration.

Somatic mutations in *PTPN11*, the gene encoding SHP2,^20^ are responsible for 35% of juvenile myelomonocytic leukemia (JMML) cases^18,21,22^ and are also implicated in other childhood cancers.^23^ WT SHP2, too, plays a pivotal role in cancer. It is essential for the survival of receptor tyrosine kinase (RTK)-driven cancer cells.^24^ It is also a central node in resistance to targeted cancer therapies,^25^ which is often caused by RTK activation through feedback loops. In addition, it mediates immune checkpoint pathways, such as programmed cell death 1 (PD-1) and signal regulatory protein α (SIRPα).^26–28^ Finally, it is involved in the development of gastric carcinoma induced by *Helicobacter pylori* .^29,30^

SHP2 plays a key role not only in cancer but also in a group of rare developmental disorders collectively referred to as RASopathies.^31^ In particular, mutations in the *PTPN11* gene are commonly associated with Noonan syndrome (NS, 50% of cases)^32,33^ and Noonan syndrome with multiple lentigines (NSML, 90% of cases).^34,35^ RASopathies are defined by features such as congenital heart defects, hypertrophic cardiomyopathy, short stature, musculoskeletal abnormalities, distinctive facial dysmorphism, and variable degrees of intellectual disability.^36^

For all these reasons, both WT SHP2 and its mutated variants are pivotal therapeutic targets for cancer and developmental disorders.^37,38^

### Role of SH2 Domains in the Allosteric Regulation of SHP2

The structure of SHP2 includes two SH2 domains (named N-SH2 and C-SH2 because they are the first and second domains from the N-terminus), followed by the PTP domain, and an unstructured C-terminal tail. In the case of SHP2, the SH2 domains mediate the association with RTKs, cytokine receptors, cell adhesion molecules, and scaffolding adaptors. They correctly localize SHP2 within the cell, recognizing sequences containing two pYs. For this phosphatase, SH2 domains also play an important role in modulating the catalytic activity of the protein. In the absence of external stimuli, SHP2 is in a closed, autoinhibited state, in which the DE loop of the N-SH2 domain (“blocking loop”) blocks the access to the active site of the PTP domain,^39^ preventing its phosphatase activity.

The association of the SH2 domains with phosphorylated sequences correlates with a conformational change in the N-SH2 domain blocking loop, which loses complementarity with the active site of the PTP domain.^40–45^ As a consequence, SHP2 activation is linked to the structural accessibility of the N-SH2 domain’s binding site, which is available only in the active state,^40,41,46^ through an allosteric regulatory mechanism that remains debated,^46–48^ with both induced fit^39^ and conformational selection models being proposed.^43,45^ Pathogenic mutations in *PTPN11* often disrupt this mechanism, leading to a constitutively active form of SHP2.

Unlike N-SH2, the accessibility of the C-SH2 domain does not seem to be influenced by this allosteric mechanism, and the binding pocket of this domain remains always accessible even in the inactive state of the protein.^46^ Even if the C-SH2 domain might not play a direct role in the activation mechanism, it participates in the recruitment of the bisphosphorylated binding partners, increasing the affinity and selectivity of the association process.^49^

### Rationale for Targeting the SH2 Domains of SHP2

Several molecules inhibiting the active site of the catalytic domain of SHP2 have been reported,^50,51^ but many of them are affected by a lack of target specificity and poor bioavailability.^52^ Even molecules with apparent binding selectivity have been demonstrated to have several off-target effects.^53^ An alternative strategy involves the development of allosteric inhibitors (also called “molecular glue”), which stabilize the autoinhibited state.^24,37,38,54–59^ To date, these compounds are undergoing clinical trials and are finding promising applications in the treatment of RTK-driven cancers^24^ and in combined therapy against drug-resistant cells.^25^ However, these inhibitors show low efficacy against hyperactive *PTPN11* mutants, because their binding site is lost in the active conformation.^37,41^

Several pieces of evidence^30,42,45,60^ showed that increased association with binding partners plays a major role in the mechanism of pathogenicity of SHP2 lesions underlying RAS/MAPK pathway hyperactivation and that proper PPIs are required for the correct function of the phosphatase. Based on these considerations, we proposed an alternative strategy focused on targeting SHP2 PPIs mediated by its SH2 domains rather than its catalytic activity. We developed a novel class of peptide-based inhibitors that disrupt SHP2 PPIs, exhibiting nanomolar affinity for N-SH2 domain, high selectivity, resistance to degradation, and strong affinity for pathogenic variants of SHP2.^10^ Due to the allosteric behavior of SHP2, these inhibitors are particularly suitable for highly activated pathogenic variants, where the N-SH2 binding site is always accessible, while they are less effective on the WT protein and on variants with minimal activation. By contrast, since the binding site of the C-SH2 domain is accessible in both activation states of the phosphatase, PPI inhibitors targeted to this domain are predicted to be effective also on the WT protein and in the variants where the autoinhibited state is prevalent. Targeting WT SHP2 is a promising strategy for treating a wide family of cancers and developmental syndromes, where hyperactivation of the pathway is caused by mutations in genes encoding downstream elements of the RAS/MAPK signaling cascade other than SHP2. In addition, should two distinct PPI inhibitors targeting specifically the N-SH2 and C-SH2 domains of SHP2 become available, the design of bisphosphorylated molecules that can simultaneously interact with both SH2 domains of the phosphatase will become possible. This approach would dramatically improve the binding affinity and selectivity of the PPI inhibitor, when compared to the isolated peptides, so that sub-nanomolar dissociation constants are conceivable. Finally, the possibility to selectively target the N- and C-SH2 domains would be an invaluable biochemical tool for investigating the role of SHP2, its PPIs, and its allosteric mechanism in the activation of different pathways, and in various pathologies.

While we were finalizing the writing of this paper, an interesting attempt to develop a sequence with high selectivity for the C-SH2 domain has been published, mainly based on Ala-scanning experiments.^61^ The final peptide had an affinity for the C-SH2 domain in the micromolar range, demonstrating that further optimization is severely needed.

### Structure and Binding Properties of SH2 Domains

The structures of SH2 domains offer critical insights into their binding properties.^4^ The first structures of SH2 domains appeared in 1992.^62–64^ Today, more than 300 three-dimensional structures of approximately 70 SH2 domains have been determined, which show a highly conserved topology,^8,65^ with α helices and β strands arranged in the order βαβββββαβ. In this paper, we will use the nomenclature due to Eck et al., 1996,^66^ where secondary structures are indicated with consecutive letters (αA and αB, βA to βG). The names of the loops are based on the secondary structure elements they connect. Each residue is then numbered consecutively within the secondary structure motifs.^40^

SH2 domains have two requirements: they must bind other proteins only when they are phosphorylated, and they must associate specifically with certain sequences only. They have two different regions dedicated to these two functions: the “pY binding cavity” and the “specificity-determining region”.^67^ In most SH2-ligand structures, the phosphopeptide sequences bind in an extended conformation and lie across the surface of the domain orthogonal to the central β sheet,^17^ composed of three antiparallel β strands flanked by two short α helices. One side of the domain, the N-terminal region, contains the αA helix and the BC loop, where the pY binding site is located. The other side, the C-terminal one, contains the αB helix, EF and BG loops, which control access to the phosphopeptide-binding region, and influence binding specificity (Figure 1).^17,68^

**Figure 1:**
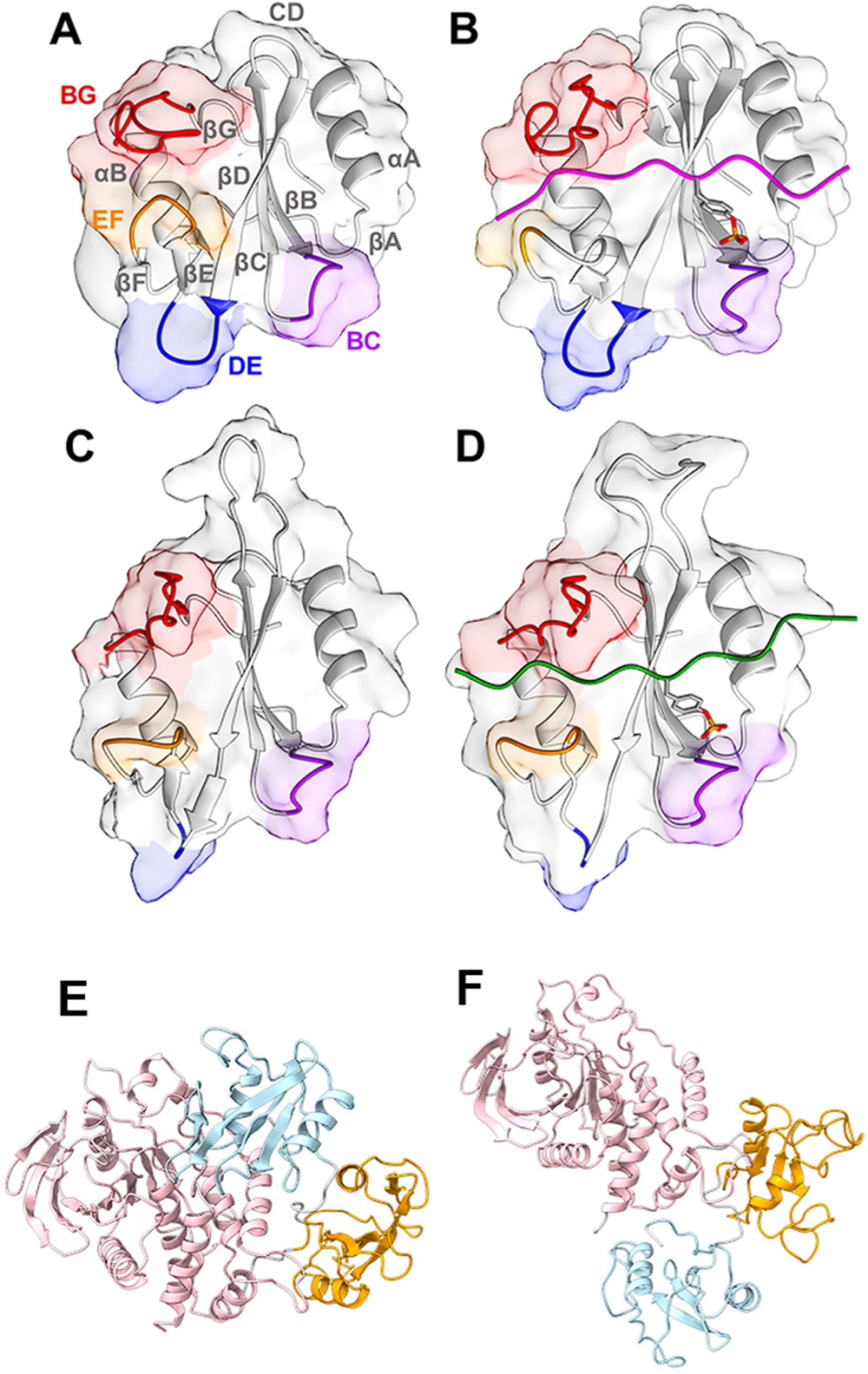
Structure of SHP2. (A) Representation of the N-SH2 domain and its secondary structure elements. (B) Representation of the N-SH2 domain complexed with PDGFR-1009. In the presence of a phosphopeptide, pY inserts in loop BC (purple), while loops EF (orange) and BG (red) control access to the groove where the C-terminal side of the peptide binds. (C) Representation of the C-SH2 domain and its secondary structure elements. (D) C-SH2 domain complexed with CagA EPIYA_D. The crystallographic structures of the N-SH2 domain (A) in the autoinhibited conformation of SHP2 and (B) when bound to a phosphopeptide differ mainly due to a rearrangement of the EF loop, which in the autoinhibited state blocks the peptide binding site of the N-SH2 domain. By contrast, in the case of the C-SH2 domain, the EF loop has the same arrangement (C) for the protein in the autoinhibited state and (D) for that bound to a phosphopeptide. Panels (E) and (F) report the structures of the whole protein in the inactive and active states, respectively, with the following color code: N-SH2 domain is light blue, the C-SH2 domain is orange, and the PTP domain is pink. PDB IDs: (A, C, E) 2SHP, (B) 4QSY, (D) 5X94, and (F) 6CRF.

The pY binding pocket is generally conserved in SH2 domains, although some exceptions exist.^6^ The most conserved residue is R­(βB5) (present in 98% of human SH2 domains), belonging to the “FLVRES” motif.^5^ It forms a salt bridge with the phosphate, which is by far the most pY-stabilizing interaction,^69^ and it is responsible for the specificity of pY binding: the tyrosine side chain is long enough to allow the interaction of the phosphate group with R, while phosphorylated S and T would be too short.^17,70^ Another generally conserved R residue is R­(αA2) (present in 82% of human SH2 domains),^5^ which interacts with the phosphate group and also makes an amino-aromatic interaction with the pY phenol ring. This residue, together with K­(βD6) (located on the other side of the pY aromatic ring with respect to R­(αA2)), generally forms a clamp around pY.^67^ Finally, the BC loop contributes to the stabilization of the peptide/domain complex, too, usually by forming hydrogen bonds (H-bonds) with the pY residue.^71^

In this study, we performed extensive MD simulations, complemented by a systematic critical analysis of different available structures and binding data, to fully characterize the dynamic and structural features of the C-SH2 domain complexes. We defined at the atomic level how the general principles common to the SH2 domain family adapt to the particular case of the C-SH2 domain, highlighting its structural specificities and determining the role of each position in the peptide sequence in the binding affinity. Our goal is to provide guidelines for the rational design of peptide or peptidomimetic inhibitors of the C-SH2 domain with high affinity and selectivity.

## Results and Discussion

### Structural Determinants of Phosphopeptide Binding to the C-SH2 Domain

#### Structural Comparison of the N-SH2 and C-SH2 Domains

The development of binders selective for C-SH2 interactions would provide molecules with potential pharmaceutical applications as well as valuable biochemical tools to clarify several debated aspects of SHP2 regulation and function. From both perspectives, it is particularly important to understand the interactions that stabilize the specific binding to C-SH2 compared with other SH2 domains and, for the development of biochemical tools, to achieve selectivity mainly with respect to the N-SH2 domain within the same protein. Since we previously determined the structural determinants for the binding affinity and selectivity of the N-SH2 domain,^47^ in this perspective, a direct comparison between the properties of the two SH2 domains of SHP2 can provide useful indications.

The different allosteric behaviors of the two domains, described above, might be related mainly to a single residue substitution in the EF loop. In the N-SH2 domain, the opening and closing of this loop is controlled by the side chain conformation of Y66­(EF1).^46,48^ In the C-SH2 domain, this residue is replaced by G182­(EF1), and the EF loop comprises three consecutive G residues, so it is possible that, without the steric hindrance of the side chain, residue EF1 is unable to modulate the conformation of the EF loop. Regarding the specificity-determining region, in the BG loop of the N-SH2 domain, K89­(BG5) and K91­(BG7) form salt bridges with the charged side chain of residues at +4 and +2 of the binding peptides, contributing to peptide binding and selectivity. Between these two K residues, an E residue is present (E90­(BG6)) and contributes to the interaction with the peptide, too.^47^ This cationic-X-cationic pattern is shared only by the N-SH2 domain of SHP2 and the C-SH2 domain of PLC-γ1. In the C-SH2 domain of SHP2, the K residues are replaced by V203­(BG4) and T205­(BG6), respectively, so the possibility of forming salt bridges involving these residues is lost. On the other hand, an E residue is still present in this C-SH2 loop (E204­(BG5), corresponding to E90­(BG6) for the N-SH2 domain). Finally, in the C-terminal part of the binding pocket, the two SH2 domains present a slightly different extent of the hydrophobic area (Figure 2B,E).

**Figure 2:**
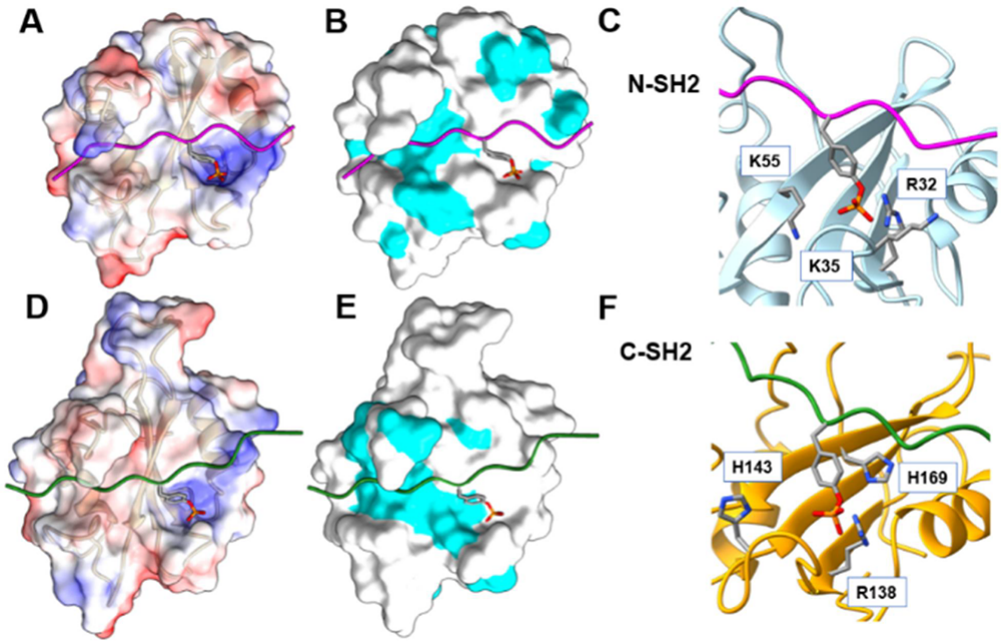
Differences in the binding sites of the N-SH2 (panels A–C) and C-SH2 domains (panels D–F). Panels (A) and (D) show the electrostatic surface potentials calculated with APBS.^73^ The color code goes from red for negative potentials (−10 kcal/(mol·e)) to blue for positive potentials (+10 kcal/(mol·e)). Panels (B) and (E) display the molecular surface of the domains, highlighting in cyan the solvent accessible surface of hydrophobic residues (A, F, L, I, P, Y, V, M, and W). Finally, panels (C) and (F) describe the pY binding pocket, highlighting residues that interact with the phosphate group of pY. PDB IDs: (A–C)4QSYand (D–F)5X94.

In the domain region interacting with the segment of phosphopeptides N-terminal to the pY residue, the C-SH2 domain contains two K residues (K120­(αA3) and K166­(βB2)),^2,71^ while the N-SH2 domain has only K35­(BC1), which intermittently forms a salt bridge with the −1 residue of the peptide (Figure 2A,D).^47^

As discussed previously, most SH2 domains form several salt bridges between the pY phosphate and cationic residues of the pY binding pocket. This is also true for the N-SH2 domain of SHP2, while the C-SH2 domain is peculiar in this respect, since it relies on critical R138­(βB5) only to form a stable ion pair with pY (Figure 2C,F). The C-SH2 domain lacks 2 out of 3 conserved residues involved in salt bridges with pY, namely, R­(αA2) and K­(βD6) (having G and M at those positions, respectively). On the other hand, the N-SH2 domain lacks R­(αA2), but retains K55­(βD6) and features a cationic residue in the BC loop (K35­(BC1)) that can form an additional salt bridge with pY. This compensatory interaction is also missing in the C-SH2 domain, where the corresponding position is occupied by Q141­(BC1), unable to form ionic interactions.

In principle, the differences described above might be partially compounded by the H residues present in the C-SH2 domain, whose protonation state at near physiological pH can vary depending on the local environment. The N-SH2 domain contains 4 H residues: H8­(βA3), H53­(βD5), H84­(αB12), and H85­(BG1), while the C-SH2 domain has 6 H residues: H114­(βA3), H116­(AA2), H132­(AB6), H143­(BC3), H169­(βD5), and H196­(αB8). Among these, H114­(βA3) and H169­(βD5) are highly conserved and are shared between both SH2 domains of SHP2. It is worth mentioning that when the C-SH2 domain forms a complex with a peptide, the pY phosphate is distant less than 1 nm (Table 1) from H143­(BC3) and H169­(βD5) (Figure 2F). Despite this proximity, the data in Table 1 suggest that the protonation of the H residues is unlikely; however, a possible role of these residues in the electrostatic stabilization of the pY in its pocket cannot be ruled out. In this regard, experimental evidence supports a functional relevance of H169: Gianni and co-workers showed that its mutation to A markedly affects the binding affinity for Gab2-derived peptides, as assessed by ITC measurements.^72^ Our p*K*_a_ analysis suggests that H169 is not protonated at physiological pH, indicating that it may contribute to peptide binding via interactions distinct from ion-pairing. The protonation states of H residues used for the simulations presented in this study are discussed in the Methods section.

**Table 1:** Protonation States and Distances from the pY Phosphate for H Residues of the C-SH2 Domain^a^

	PROPKA3.1		H++		distance (nm)
	C-SH2/peptide complexes	C-SH2	C-SH2/peptide complexes	C-SH2	C-SH2/peptide complexes
H114 (βA3)	6.2 ± 0.4	6.3 ± 0.4	6.1 ± 0.6	5.9 ± 0.6	1.4 ± 0.1
H116 (AA2)	6.5 ± 0.1	6.5 ± 0.1	6.7 ± 0.3	6.4 ± 0.3	1.2 ± 0.2
H132 (AB6)	6.5 ± 0.5	6.5 ± 0.3	5.0 ± 1.0	5.0 ± 1.0	2.0 ± 0.1
H143 (BC3)	6.4 ± 0.3	6.5 ± 0.3	7.6 ± 0.7	6.5 ± 0.4	0.8 ± 0.1
H169 (βD7)	4.5 ± 0.9	5.3 ± 0.4	4.0 ± 2.0	4.0 ± 1.0	0.7 ± 0.1
H196 (αB8)	6.0 ± 0.2	6.1 ± 0.1	6.9 ± 0.4	6.8 ± 0.3	2.0 ± 0.2

a The p*K*_a_ values were computed using PROPKA3.1 (first column) and H++ (second column), both for the C-SH2/peptide complexes and for the C-SH2 domain alone. The last column refers to the distance between the center of mass of the phosphate group of pY and the aromatic ring of the H residues of the domain. The reported values correspond to averages and standard deviations obtained using the available crystallographic (Eck, 5X94, 5DF6, 5X7B) and NMR (6R5G) structures.

A final structural difference between the two domains is in the length of the CD loop (12 vs 3 residues in the C-SH2 and N-SH2 domains, respectively) (Figure 1).

Overall, the dissimilarities discussed in this section can provide indications of the different allosteric behavior and binding properties of the two domains.

#### Sequence Selectivity of the C-SH2 Domain

Several data are available regarding the binding selectivity of the C-SH2 domain, deriving from the sequences of natural binders, quantitative peptide binding studies, and high-throughput qualitative peptide library experiments.

Several proteins that interact with SHP2 through their SH2 domains have been identified in earlier studies.^74–83^ In addition, several peptide ligands of the C-SH2 domain have been reported in the literature.^49,61,75,76,84–87^ Quantitative binding studies have been conducted for some of these sequences and for artificial peptides. Table 2 summarizes the phosphorylated sequences for which a high binding affinity for the C-SH2 domain of SHP2 has been reported (sub-micromolar dissociation constants). With a few exceptions only, a consensus pattern can be defined, with the following preferred residue types: hydrophobic at positions +1 and +3, aromatic at +5, and anionic at −1.

**Table 2:** Sequences with a Dissociation Constant for the C-SH2 Domain of SHP2 in the Nanomolar Range^a^

protein	pY	–7	–6	–5	–4	–3	–2	–1	0	+1	+2	+3	+4	+5	+6	+7	+8	*K*_*d*_ C-SH2 SHP2 [nM]	*K*_*d*_ N-SH2 SHP2 [nM]	*K*_*d*_ C-SH2 SHP1 [nM]	*K*_*d*_ N-SH2 SHP1n[nM]	ref
PD-1 ITSM	248				*E*	Q	T	*E*	pY	**A**	T	**I**	**V**	**F**	**P**			13	170	^b^	^b^	^49^
Gab1	659		**A**	*D*	*E*	*R*	**V**	*D*	pY	**V**	**V**	**V**	*D*	Q				27	n.a.	n.a.	n.a.	^85^
IRS-1	1222					**L**	S	T	pY	**A**	S	**I**	N	**F**	Q	*K*		110	900	n.a.	n.a.	^87^
PDGFR	1009		*D*	T	S	S	**V**	**L**	pY	T	**A**	**V**	Q	**P**	N			240	n.a.	n.a.	n.a.	^86^
IRS-1	895				S	**P**	G	*E*	pY	**V**	N	**I**	*E*	**F**	G	S		310	390	n.a.	n.a.	^87^
gp130	757	S	T	**A**	S	T	**V**	*E*	pY	S	T	**V**	**V**	*H*	S	G		550	1̇200	n.a.	n.a.	^84^
artificial							T	**I**	pY	**A**	T	**I**	**L**	N	**B**	*K*	R	600	3̇900	6̇400	2̇400	^75^
artificial					**A**	**A**	**L**	N	pY	**A**	Q	**L**	**X**	**F**	**P**			930	70	9̇800	170	^76^
artificial					N	N	**I**	T	pY	S	**L**	**L**	**X**	**F**	**P**			980	200	2̇100	360	^76^

a PD-1: programmed cell death protein 1; Gab1: GRB2-associated binding proteins 1; IRS-1: insulin receptor substrate 1; PDGFR: platelet-derived growth factor receptor; gp130: glycoprotein 130. B indicates beta-alanine, X norleucine. Aromatic, hydrophobic, cationic, and anionic residues are reported in bold, underlined bold, italic, and underlined italic, respectively. pY numbers and sequences for IRS-1 refer to the rat protein, for gp130 to the murine enzyme. Association of Gab1 and PDGFR peptides was measured with the tandem SH2 domains. Experiments on the gp130 peptide demonstrated that the minimal sequence −2 to +5 retains the whole binding affinity.^84^ For PD-1, discrepant *K*_*d*_ values have been reported: 13 nM,^49^ 100 nM,^88^ and 1.6 μM.^89^

b Experiments using the whole PD-1 protein, with the ITIM Tyr residue mutated to F, so that only the ITSM Tyr can be phosphorylated, indicated a low binding selectivity: the affinities for C- and N-SH2 domains of SHP2 and SHP1 were: 100 (C-SH2 SHP2), 140 (N-SH2 SHP2), 1700 (C-SH2 SHP1), and 80 nM (N-SH2 SHP1).^88^

In parallel, the sequence selectivity of the C-SH2 domain of SHP2 has also been analyzed by high-throughput studies with phosphopeptide libraries, with results summarized in Table 3. Based on the findings from these studies, binding preferences were identified at positions ranging from −3 to +6. A distinct preference for hydrophobic residues at positions +1 and +3 emerges, consistent with the sequences listed in Table 2, for polar residues at position +2 and for anionic and hydrophobic residues at position −3. Other positions, such as +4, +5, and +6, appear to be less clearly defined, as the presence of either cationic or aromatic residues is suggested.

**Table 3:** Motifs Determined from Peptide Library Studies^a^

–3	–2	–1	0	+1	+2	+3	+4	+5	+6	Reference
			pY	**A** ( **V** TS)		**I** ( **LV** )		**WF**		^84^
	T **VIY**		pY	**A** (ST **V** )		**I** ( **V** )				^75^
			pY				**W** *RH* **Y**	*HR*	*HR* **Y**	^76^
*E* **M**	*E* **V**		pY	**VALI**	N	**LVI**				^42^
				**L**	N	**L**				^90^
	T **VI**			**A** ST		**LVI**				^91^

a The sequence positions investigated in each study have a thicker border. Aromatic, hydrophobic, cationic, and anionic residues are reported in bold, underlined bold, italic, and underlined italic, respectively.

The amino acid preferences observed in high-affinity peptide sequences and peptide library studies are summarized graphically in Figure 3A,B, respectively.

**Figure 3:**
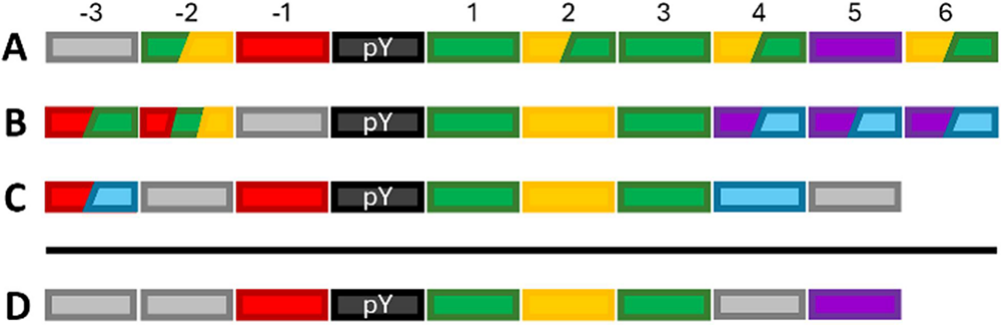
Schematic representation of amino acid preferences at each position, based on high-affinity natural binders (A), peptide libraries (B), MD simulations (C). Combined pattern (D) based on residues consistently found across A–C. Positions lacking agreement were left unassigned. Color code: hydrophobic (green), aromatic (purple), polar (yellow), anionic (red), cationic (light blue), unassigned (gray), and phosphotyrosine (black).

#### Structural and Dynamical Analysis of the C-SH2 Domain

#### Structures of C-SH2/Phosphopeptide Complexes and MD Simulations

Table 4 lists the available experimental structures for complexes of the C-SH2 domain with phosphopeptides. They include natural sequences from the PD-1 ITSM motif (PDB ID:6R5G), PDGFR (Eck), CagA EPIYA-D (PDB ID:5X94), CagA EPIYA-C (PDB ID:5X7B), and TXNIP (PDB ID: 5DF6). In the following sections, these structures are analyzed with respect to the interactions determining binding affinity and selectivity. However, X-ray structures provide a static picture, which does not offer any indication of possible conformational transitions or of the stability of the interactions. To gain this deeper understanding, we conducted 1.2 ms simulations of seven different C-SH2 domain complexes. As shown in Table 4, most simulations focused on residues −3 to +5. Obviously, minimizing the peptide sequence is desirable in view of possible therapeutic applications of the peptides, but this choice was further supported by two previous studies: (i) an investigation of the gp130 peptide showed that the minimal sequence −2 to +5 is sufficient to retain the full binding affinity;^84^ (ii) our previous library study indicated specific preferences also for residue −3.^42^ In one case (PD-1), we also tested the sequence −4 to +6 (indicated as PD-1_FULL) for comparison with the NMR data^49^ obtained using the same peptide. In addition, we analyzed sequences from natural ligands (PD-1, Gab1, and IRS-1) and tested the effects of various amino acid substitutions that were selected based on the results obtained from library screening studies (Table 3): E was introduced at −3 or −2,^42^ R at +4,^76^ or W at +5.^84^

**Table 4:** C-SH2/Peptide Complexes (Experimental and Simulated)^a^

method	ID	–7	–6	–5	–4	–3	–2	–1	0	+1	+2	+3	+4	+5	+6	+7	relative *K* *_d_*	reference
NMR	6R5G (PD-1 ITSM)				*E*	Q	T	*E*	pY	**A**	T	**I**	**V**	**F**	**P**		1	^49^
X-ray	Eck (PDGFR)					S	**V**	**L**	pY	T	**A**	**V**	Q	**P**	N	*E*	*	
	5X94 (CagA EPIYA-D*)*		**A**	S	**P**	*E*	**P**	**I**	pY	**A**	T	**I**	*D*	**F**	*D*		110	^29^
	5DF6 (TXNIP)	*K*	**F**	**M**	**P**	**P**	**P**	T	pY	T	*E*	**V**	*D*				3̇000	^68^
	5X7B (CagA EPIYA-C)		**v**	s	**p**	*e*	**P**	**I**	pY	**A**	T	**I**	*D*	*D*	**L**		4̇654	^29^
MD	PD-1_FULL				*E*	Q	T	*E*	pY	**A**	T	**I**	**V**	**F**	**P**		1	^49^
	PD-1					Q	T	*E*	pY	**A**	T	**I**	**V**	**F**			*	
	GAB1					*R*	**V**	*D*	pY	**V**	**V**	**V**	*D*	Q			*	
	IRS-1					**L**	S	T	pY	**A**	S	**I**	N	**F**			*	
	PD-1_E-3					*E*	T	*E*	pY	**A**	T	**I**	**V**	**F**			-	
	PD-1_R+4					Q	T	*E*	pY	**A**	T	**I**	*R*	**F**			-	
	PD-1_W+5					Q	T	*E*	pY	**A**	T	**I**	**V**	**W**			-	
	IRS-1_E-2					**L**	*E*	T	pY	**A**	S	**I**	N	**F**			-	

a Aromatic, hydrophobic, cationic, and anionic residues are reported in bold, underlined bold, italic, and underlined italic, respectively. Residues in lowercase were not resolved in crystallographic structures. Eck indicates the undeposited structure determined in Eck et al.^66^ References indicated in the last column concern data on relative dissociation constant (*K*_*d*_) values, which were normalized to that of PD-1 ITSM. Asterisks indicate truncated simulated sequences; the full-length affinity has been reported in Table 2. Dashes indicate sequences whose affinity has not been measured.

The NMR structure (PDB ID: 6R5G) of the C-SH2/PD-1 ITSM complex,^49^ which has the highest experimentally determined binding affinity (Table 2) served as the starting point for our simulations. Since no structural data were available for complexes with Gab1, IRS-1, or the artificial sequences, we adapted the NMR model to these simulations by making the necessary substitutions in the peptide ligand, as described in the Methods section.

#### The −1 to +5 Phosphopeptide Region Interacts Stably with the Domain

In all simulations, the peptides remained firmly bound to the domain for the entire trajectory, but their N-terminal portions displayed significant mobility. This behavior is effectively illustrated by the per-residue averaged root-mean-square fluctuations (RMSF) of the peptide atom positions (Figure 4). In all cases, RMSF values were lower than 2 Å for residues 0 to +4, and in most trajectories, the stable stretch extended from −1 to +5. By contrast, residues preceding −1 had RMSF values greater than 2 Å in all simulations. The RMSF values obtained from the Debye–Waller factors observed in crystallographic structures and the variability seen in NMR solution structures were generally consistent with a low mobility of the −1 to +4 region. More importantly, the overlap of experimental structures reported in Figure 4 shows that the N-terminal peptide sequence experiences significant conformational heterogeneity among the different structures. These findings are in apparent contrast with the results of peptide library studies, indicating that N-terminal residues can significantly influence binding affinity (Table 3), and with the observation that N-terminal peptide residues are resolved in most of the X-ray structures (Table 4). The latter finding could be caused by the crystal field. Confirming this hypothesis, Figure 4D shows that structures containing a peptide with a longer N-terminal stretch have significant crystalline contacts; such interactions may artificially stabilize this segment and reduce its apparent flexibility. Nonetheless, the flexibility in the N-terminal region observed in the MD simulations and the results of peptide library studies become coherent if transient ion-pair interactions between the bound peptides and the C-SH2 domain are taken into account. Such interactions can become evident through MD simulations, which effectively capture both the conformational flexibility and the propensity for transient interactions in these regions (see below).^92^

**Figure 4:**
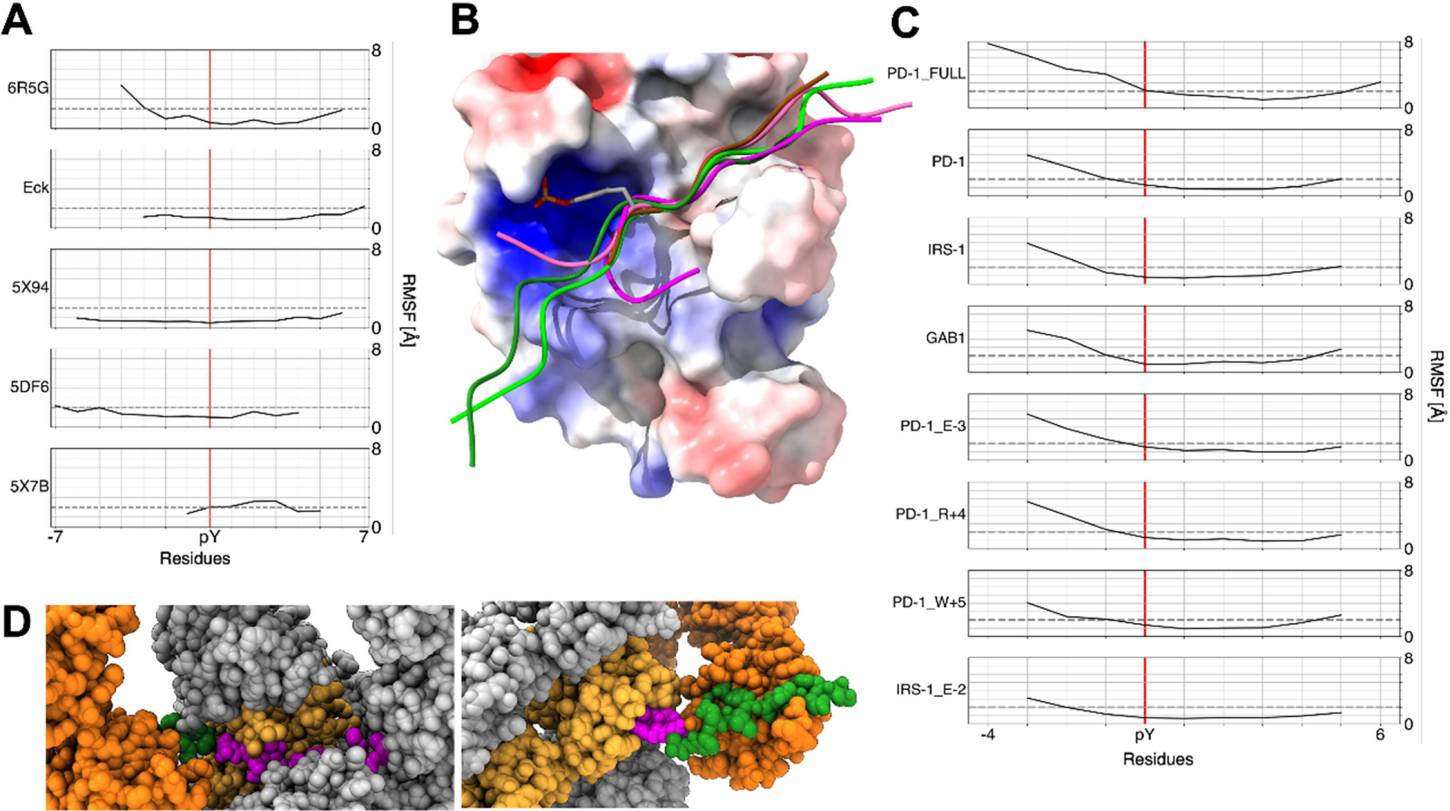
Mobility of bound peptides. (A) Backbone RMSF from crystallographic structures and NMR data. The dashed gray horizontal line corresponds to an RMSF value of 2 Å. (B) Structural representation of the conformational heterogeneity of the phosphopeptide N-terminal segment among experimental structures: 6R5G: magenta, Eck: pink, 5X94: light green, 5DF6: dark green, 5X7B: brown. The surface of the C-SH2 domain is colored based on the electrostatic potential (from red for negative potentials (−10 kcal/(mol·e)) to blue for positive potentials (+10 kcal/(mol·e)). (C) Backbone RMSF from MD trajectories. The dashed gray horizontal line corresponds to the RMSF value of 2 Å. (D) Crystal contacts involving the peptide in 5DF6 (left) and 5X94 (right) structures. The atomic structure is colored light orange (C-SH2 domain) and magenta (peptide). Among the symmetry-related copies, the replica showing contacts with the N-terminal region of the peptide is highlighted in dark orange (C-SH2 domain) and green (peptide). Other copies are shown in gray.

#### The C-Terminal Region of the Peptides Is Mostly in an Extended Conformation

Figure S1 shows the Ramachandran plots of the peptide backbone in the X-ray and NMR structures and in the MD simulations. In the crystallographic structures, the peptides consistently adopted an elongated conformation for all residues.^93,94^ A higher variability was found among the different NMR conformations (6R5G), but the central region (residues −2 to +3) was mostly in an extended structure, too. In simulation studies, the central segment of the peptide (comprising residues from 0 to +3) maintained an extended conformation, and residues at positions +4 and +5 were often found in an extended structure, too. By contrast, the N-terminal segment (residues −4 to −1) exhibited greater flexibility, exploring various areas of the Ramachandran plot.

The extended conformation of the C-terminal region of the ligands is stabilized by a network of H-bonds between the peptide backbone and the C-SH2 domain (Table 5). These bonds mostly involve peptide residues +1, +2, and +4, and protein residues H169­(βD5), T205­(BG6), and V203­(BG4), respectively. These interactions were observed in all the crystallographic structures and persisted during the simulations. The corresponding H-bonds were previously observed also in simulations of peptide complexes with the N-SH2 domain.^47^ In addition, in the NMR data and with different stabilities, in several simulations, the residue at position +1 formed an H-bond with M171­(βD7). Stable H-bonds involving residues in the N-terminal region of the peptide were not observed, neither in the structures nor in the simulations, coherently with comparatively high flexibility.

**Table 5:** H-Bonds between the Peptide Backbone and the C-SH2 Domain^a^

		+1		+2	+4
method	ID	N H169^O^ (βD5)	O M171^N^ (βD7)	O T205^N^ (BG6)	N V203^O^ (BG4)
NMR (%)	6R5G	**60**	**50**	-	10
X-ray (Å)	Eck	**2.7**	-	**2.9**	**2.9**
	5X94	**2.7**	-	**3.0**	**2.9**
	5DF6	**2.9**	-	**2.6**	**2.9**
	5X7B	**2.7**	-	**3.1**	-
MD (%)	PD-1_FULL	45	9	13	**70**
	PD-1	**51**	-	14	**72**
	IRS-1	49	-	20	**59**
	GAB1	**51**	27	8	46
	PD-1_E-3	**54**	20	13	**69**
	PD-1_R+4	45	**52**	17	**68**
	PD-1_W+5	**50**	5	14	**71**
	IRS-1_E-2	35	-	21	**69**

a H-bond stability was characterized based on interatomic distances (in Å) for X-ray structures, or % persistence values for MD simulations and NMR structures. H-bonds were considered stable for distances ≤ 3.5 Å (in X-ray structures) or persistence ≥ 50% (in MD simulations and NMR structures) and are highlighted in bold. Dashes indicate that the H-bond is not formed (in X-ray structures) or that it is present for less than 5% (in MD simulations or NMR structures). Data are reported only for H-bonds that were stable in at least one of the simulations or structures. The numbers reported in the first line refer to the peptide residues, counted with respect to the pY position. In the second line, the C-SH2 residue is indicated, and the backbone atoms involved in H-bonds are shown as superscripts.

#### Phosphotyrosine Interactions

As discussed in the introduction, phosphopeptide/SH2 domain complexes are also strongly stabilized by the interactions of the pY residue with its binding site.

The C-SH2 domain is characterized by the presence of a single cationic residue in the pY pocket, i.e., the highly conserved and crucial R138­(βB5). The R138-pY salt bridge was consistently observed in all the experimental structures and throughout all simulations (Table 6); the only exception in Table 6, 1 out of 10 NMR structures in 6R5G presents a distance a bit larger than the cutoff (0.45 nm, with respect to a cutoff set at 0.40 nm).

**Table 6:** H-Bonds and Salt Bridges between pY and the C-SH2 Domain^a^

		H-bonds				salt bridges
		S140 (βB7)	Q141 (BC1)	S142 (BC2)		R138 (βB5)
method	ID	side chain Oγ	backbone N	side chain Oγ	backbone N	
NMR (%)	6R5G	20	10	40	10	**90**
X-ray (Å)	Eck	-	**2.8**	-	**2.9**	**2.9**
	5X94	-	**3.4**	-	3.8	**3.4**
	5DF6	-	**3.0**	-	**3.1**	**3.4**
	5X7B	-	**2.9**	-	**3.0**	**3.5**
MD (%)	PD-1_FULL	22	9	30	8	**100**
	PD-1	49	5	47	-	**99**
	IRS-1	**52**	-	49	-	**99**
	GAB1	25	13	35	15	**99**
	PD-1_E-3	40	6	38	8	**99**
	PD-1_R+4	**55**	8	**56**	18	**98**
	PD-1_W+5	43	8	48	-	**100**
	IRS-1_E-2	**85**	-	**79**	-	**99**

a H-bond and salt-bridge stability was characterized based on interatomic distances (in Å) for X-ray structures, or % persistence values for MD simulations and NMR structures. H-bonds were considered stable for distances ≤ 3.5 Å (in X-ray structures) or persistence ≥ 50% (in MD simulations and NMR structures). Salt bridges were considered stable for distances ≤ 4.0 Å (in X-ray structures) or persistence ≥ 50% (in MD simulations and NMR structures). Stable bonds are highlighted in bold. Dashes indicate that the bond is not formed (in X-ray structures) or that it is present for less than 5% (in MD simulations). Data are reported only for H-bonds that were stable in at least one of the simulations or structures.

The pY residue is additionally stabilized by a network of H-bonds,^71^ which usually involve S­(βB7) (present in 88% of human SH2 domains) and residues of the BC loop.^67^ Our data confirm these interactions: the phosphate group of pY was bound to the side chains of S140­(βB7) and to S142­(BC2) in the NMR structure and in the simulations, but not in the crystallographic structures (Table 6). The backbone of the BC loop (Q141­(BC1) and S142­(BC2)) also contributed to the formation of H-bonds with pY (Table 6). These interactions are similar to those formed by the N-SH2 domain, which, however, has an additional very stable side chain H-bond formed by residue T42­(βC3).^47^ The corresponding amino acid in the C-SH2 domain is V148­(βC3), so that this interaction is not possible.^66^ Despite the spatial proximity of H143­(BC3) and H169­(βD5) to the pY phosphate group (less than 1 nm, see Figure 2F and Table 1), no H-bonds were observed. However, this finding does not exclude a functional or structural role in phosphate recognition.

Overall, our data indicate that the pY residue is stabilized in the C-SH2 binding pocket by fewer interactions than those observed in the N-SH2 domain. These differences could help to explain the distinct preferences for different pY mimics recently observed for the two domains.^61^ For studies aimed at designing selective peptides targeting C-SH2 over other SH2 domains, this peculiarity could be exploited by incorporating suitable nondephosphorylatable pY analogues.

#### “Selectivity-Determining Region”: Residues +1 and +3 Insert in Hydrophobic Pockets

Based on the interactions in this selectivity-determining region (i.e., where residues C-terminal to the pY bind), the SH2 domains have been classified into three classes.^3,67,95,96^ The C-SH2 domain of SHP2 belongs to the type II, called “open groove”, or “PLC-γ1-like”.^3^ Typically, in this class of domains, the peptide binds perpendicular to the central β-sheet, where residues C-terminal to the pY, characterized by the pY-hydrophobic-X-hydrophobic pattern, fit in a long hydrophobic groove extending up to +5 and are delimited by the EF and BG loops.

The solvent accessible surface (SAS) of peptide side chains enabled us to quantitatively analyze apolar interactions during the simulations and in the experimental structures (Figure 5). Residues +1 and +3 were consistently nestled within the hydrophobic groove, while residues +2 and +4 faced the solvent. This pattern was interrupted in correspondence with residue +5, which was exposed to the solvent. Residues +1 and +3 interact with hydrophobic amino acids that line the groove. In particular, residue +1 interacts with V170­(βD6), L210­(βG3), the methyl groups in the side chains of T168­(βD4), and the aliphatic groups in the E204­(BG5) side chain. Residue +3 always interacts with V170­(βD6), V181­(βE4), G182­(EF1), G183­(EF2), M202­(BG3), and L210­(βG3) and with the methylene groups in the side chain of E204­(BG5). In GAB1, PD-1_R+4, and PD-1_W+5 simulations, it can also interact with Y197­(αB9).

**Figure 5:**
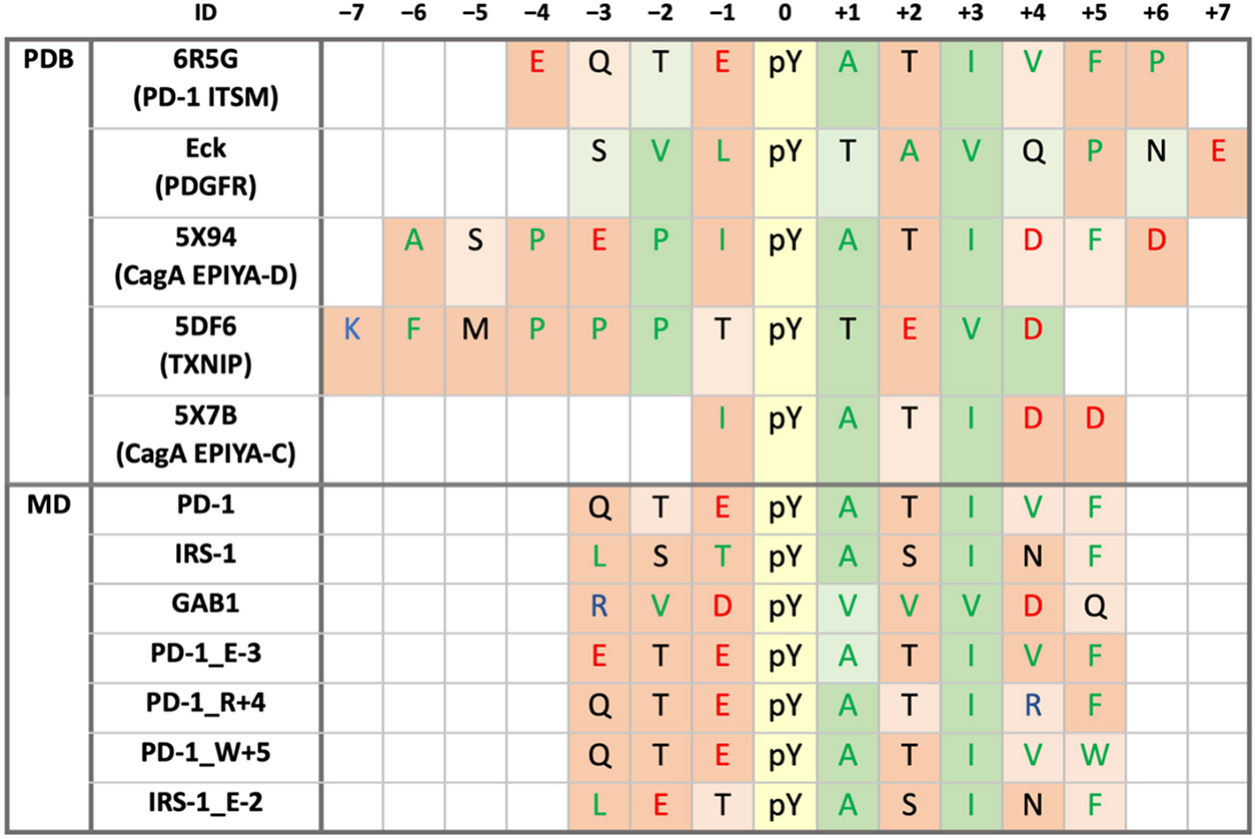
Solvent exposure of the phosphopeptide residues. Except for pY, residues are colored in green or orange for solvent accessible surface lower or higher than 50%. Values are divided into four intervals: lower than 35% (dark green), between 35 and 50% (light green), between 50 and 65% (light orange), and greater than 65% (dark orange). For MD simulations, the average value was considered. Hydrophobic, anionic, and cationic residues are indicated by green, red, or blue letters, respectively.

These findings are consistent with the strong prevalence of apolar amino acids at positions +1 and +3 observed in peptide library studies (Table 3). The solvent exposure of residue +5 is consistent with the lack of a specific preference for a hydrophobic side chain at position +5, which is a peculiarity of the C-SH2 domain in the type II family.

In the simulations, the N-terminal peptide residues were all exposed to the solvent, paralleling the significant mobility of this region. Even when V was present at −2, as in the GAB1 simulation, its side chain remains solvent-exposed. The analysis of the experimental structures was mostly in agreement with the simulation results, except for the −2 residue, which was not solvent-exposed in the X-ray structures, possibly due to crystal field effects in this region, as already discussed above. Indeed, in the NMR structures, the polar T residue present at position −2 was more solvent-exposed than the hydrophobic side chains at the corresponding positions in the crystallographic structures. On the other hand, the presence of a hydrophobic residue at −2, as suggested by library studies (Table 3), is possibly helpful to allow the proper formation of the pY binding pocket, due to the lack in the C-SH2 domain of the conserved RαA2 residue that generally forms a wall of the pY binding cavity.^67^

In this context, data from our simulations clearly indicate that residues at −2 and −3 play a critical role in the modulation of the solvent exposure of the pY side chain. As shown in Table 7, solvent exposure values calculated over peptide residues −1 to +5 are significant, with values across the simulations, up to roughly one-third of the value of an isolated pY. The N-terminal extension to include residue −2 results in a sequence-dependent reduction in solvent exposure (down to a range of 20–27%). Interestingly, a further extension to residue −3 amplifies this “cage” effect, with a final exposure range of 16–22%. These results suggest that modifying the N-terminal sequence could be a valid strategy to further reduce pY solvent exposure and stabilize the binding environment.

**Table 7:** pY Solvent Exposure upon N-terminal Extension of the Peptide^a^

		solvent exposure		
method	ID	–1 to 5	–2 to 5	–3 to 5
MD (%)	PD-1	29	25	22
	IRS-1	29	25	21
	GAB1	22	20	17
	PD-1_E-3	28	21	19
	PD-1_R+4	23	20	16
	PD-1_W+5	29	23	17
	IRS-1_E-2	29	27	19

a Solvent exposure of the pY side chain during MD simulations. The percentage of solvent exposure is calculated in the presence of peptide residues −1 to +5 (third column), −2 to +5 (fourth column), and −3 to +5 (last column).

#### Interactions of Solvent-Exposed Residues

Residues +2, +4, and +5 are solvent-exposed (Figure 5), but interactions with the EF and BG loops that delimit the binding groove of the C-terminal peptide portion are possible (Table 8). Stable H-bonds were indeed observed between T+2 of the PD-1 peptide and T205­(BG6) in the NMR structure and in the PD-1_W+5 simulation, between R+4 and T205­(BG6) in the PD-1_R+4 simulation, and between W+5 and G182­(EF1) in the PD-1_W+5 simulation.

**Table 8:** H-Bonds and Salt Bridges between Peptide Side Chains and the C-SH2 Domain^a^

									salt bridges				
		H-bonds							–4	–3		–1	
method	ID	–3	–2	–1	+1	+2	+4	+5	K166 (βD2)	K120 (αA3)	E123 (αA5)	K120 (αA3)	K166 (βD2)
NMR (%)	6R5G	-	T^Oγ^-E123^Oε2^:10 (αA5)	-		T^Oγ^-T205^N^:10 (BG6)			**E:50**			-	E:10
X-ray (Å)	Eck	H_2_O bridge			-								
	5X94	-				-	-						
	5DF6			-	H_2_O bridge	H_2_O bridge	-						
	5X7B					-	-	-					
MD (%)	PD-1	-	-	*		-						E:7	E:11
	IRS-1		S^Oγ^-E123^Oε1^:7 (αA5)			-	-	-					
	GAB1	*		-			-	Q^Oε^-H196N^ε^:8 (αB8)		-	R:15	-	-
	PD-1_E-3	*	-	*		-				E:14		E:11	E:11
	PD-1_R+4	-	T^Oγ^-S142^Oγ^:8 (BC2)	*		-	R^Nη^-T205^O^:11 (BG6)	-				E:5	-
	PD-1_W+5	-	-	*		T^Oγ^-T205^Oγ1^: 6 (BG6)		W^Nε^-G182^O^:17 (EF1) W^Nε^-G182^C^:8 (EF1)				E:14	E:15
	IRS-1_E-2		-	-		-	-						

a Distances (Å) and % persistence are reported for X-ray structures and MD simulations, respectively. Peptide residues are numbered with respect to the pY. Stable bonds (distance ≤ 3.5 Å for H-bonds and ≤ 4.0 Å for salt bridges in X-ray structures or persistence ≥ 50% in MD simulations and NMR structures) are highlighted in bold. Ion-pair distances were calculated between the centers of mass of the charged groups of the residues. Dashes indicate that the H-bond is not formed in X-ray structures and that it is present for < 5% in MD simulations; asterisks indicate that the H-bond is not reported because the same interaction was considered as an ion pair. Peptide residues in +3 are omitted because they cannot form H-bonds in any of the studied complexes. In the Eck structure, an A side chain is present instead of K at position 166 of the domain. For H-bonds, the convention used shows the peptide residue with the involved atom in superscript, followed by the protein residue (with secondary structure-based numbering in parentheses) and its relevant atom in superscript.

SH2 domains of SHP2 are one of the few exceptions where, based on peptide library studies (Table 3), residues N-terminal to pY are important for binding.^17,96^ Consistent with this finding, and despite the relatively higher mobility of the N-terminal segment, compared to the C-terminal side (Figure 4), H-bonds between the domain and a polar side chain (S or T) at position −2 were observed in the NMR structures and in IRS-1 and PD-1_R+4 simulations. In addition, several ion-pair interactions were observed (Table 8). MD simulations of PD-1 and its analogues show that the E residue at position −1 can make a salt bridge with two different K residues of the domain: K120­(αA3) and K166­(βD2) (Figure S2). The lifetimes of these interactions are shorter than those observed for residues in the C-terminal region of the peptides, making this region more flexible. However, their presence, clearly detectable in both the structures and our simulation, likely contributes to stabilizing the overall peptide/domain interaction.

In contrast to PD-1-derived sequences, GAB1 presents a D residue at −1, and the shorter side chain does not allow salt-bridge formation with either of the two K residues in the C-SH2 domain (Figure S2). However, the GAB1 peptide can create another interaction between *R*-3 and E123­(αA6) of the domain, a residue conserved in 61% of the human SH2 domains^68^ (Figure S3C,D).

Regarding the substitutions introduced in the PD-1 sequence, based on our previous library studies (Table 3),^42^ replacing Q-3 with E enables the formation of an additional salt bridge with K120­(αA3) (Figure S3A,B). In the X-ray structure 5X94, an E-3 residue is also present, and the charged groups are at a distance of 4.8 Å. Although this value is too large for a salt bridge, a strong electrostatic interaction is confirmed, and this substitution seems to give a favorable contribution to binding.

Overall, these results suggest that introducing a charged residue at position −3 could enhance the binding affinity of the C-SH2 domain (Figure 3C). Of note, Kiani et al.,^61^ on the basis of Ala-scan, concluded that the N-terminal region of the peptide is not important for selectivity. However, only substitutions for A were considered in this case (e.g., Q-2A).

Although a salt bridge is not formed, the interaction between R at position +4 of PD-1_R+4 and E204­(BG5) could contribute to the association event; Figure S4 shows that distances between 6 and 8 Å are stably retained between the charged groups of the two residues, producing an electrostatic attraction that could contribute to stabilizing the complex. In addition, the presence of a cationic residue at position +4 makes PD-1_R+4 a particularly interesting candidate for a selective inhibitor with respect to the SHP2 N-SH2 domain, as a high affinity to the N-SH2 domain would require an anionic residue at this position.^47^ Interestingly, an anionic residue at position +2 is required for strong binding to the N-SH2 domain, too.^10,47^ Based on this observation, we hypothesize that introducing a cationic residue at this position could further enhance selectivity with respect to N-SH2; a favorable effect on affinity cannot be ruled out for the spatial proximity of E204.

Even if this substitution is not suggested by peptide library studies, the spatial arrangement of E204 indicates that it might be favorably oriented to interact electrostatically with solvent-exposed cationic residues in +2.

The amino acid preferences predicted based on our structural and dynamical analysis are graphically summarized in Figure 3C. The overall consensus pattern derived from the different sources (i.e., from Figure 3 A–C) is reported in Figure 3D.

## Conclusions

This work provides a detailed analysis of the structural determinants governing the binding affinity and selectivity of the C-SH2 domain of SHP2 and proposes this domain as a promising pharmacological target. Our integrated analysis, based on available binding studies, peptide library, and structural data, and our MD simulations, provides an in-depth understanding of the binding features required for this domain. Our findings show that the −3 to +5 region of the peptide is the minimal required for effective binding to the C-SH2 domain. In particular, they reveal how residues from −1 to +5 contribute to stable interactions as they are tightly bound to the domain, with residues from 0 to +3 consistently adopting an extended conformation. In contrast to other SH2 domains, the pY is stabilized in its pocket by a single electrostatic interaction, and several H-bonds further contribute to its stability. This evidence suggests that employing suitable nondephosphorylatable pY-mimicking residues could provide an uncommon strategy to enhance the selectivity of binders for this domain over other SH2 domains. Side chains of residues in the C-terminal portions of the bound peptides show an alternating exposed/buried pattern, with hydrophobic residues at positions +1 and +3, interacting with the apolar side chains of the domain binding groove. It is worth noting that the presence of hydrophobic residues at these positions is consistent with all the sequences derived from the different studies analyzed (Figure 3). The presence of a polar residue at position +2 is consistent with results from peptide library studies and is further supported by the solvent exposure at this position observed in our simulations. Consistent with most SH2 domains, the main driving force for binding of the C-terminal peptide segment is the hydrophobic effect, with stabilization provided by backbone H-bonds, even if other interactions contribute to defining the specificity of this domain. For instance, introducing cationic residues at position +4 can promote an increase in binding selectivity to the C-SH2 domain with respect to N-SH2. At the +5 position, the presence of a cationic or aromatic residue is observed, as supported by both high-affinity sequences and library data. Our simulations suggest that the substitution of F with W does not significantly alter the binding mode, indicating some degree of tolerance between the aromatic side chains. The association of the more mobile N-terminal part is essentially guided by intermolecular ion pair interactions, further contributing to binding specificity. In addition, transient but relevant interactions, highlighted by our MD simulations and not detected in crystallographic structures, may strongly contribute to improving binding stability. Due to this evidence, the presence of charged residues at position −3, along with anionic ones at −1, may favor phosphopeptide binding to the C-SH2 domain. The nature of the residue at −2 remains unclear. High-affinity ligands show hydrophobic or polar amino acids at this position, while peptide libraries show a mixed profile with the presence of hydrophobic, anionic, polar, and aromatic residues. From a pY coordination perspective, a hydrophobic side chain may help compensate for the absence of R­(αA2), potentially contributing to the formation of the binding pocket. In our simulations, the presence of an anionic residue at −2 did not provide any clear advantage, while a polar residue was observed to give additional stability by forming H-bonds. This aspect will be further investigated in future work. In agreement with the recent findings by Kiani et al.,^61^ our data highlight the significance of positions +4 and +5 in conferring selectivity for C-SH2 over N-SH2. Overall, our work reveals the structural determinants responsible for sequences achieving high affinity and selectivity for the C-SH2 domain of SHP2.

## Methods

Experimental structures were taken from X-ray (PDB ID: 5DF6, 5X7B, 5X94, Eck) and NMR (PDB ID: 6R5G) structures. For the PD-1_FULL simulation, initial atomic coordinates were taken from NMR data of 6R5G (model 1). The other simulated sequences (Table 4) were obtained by substituting, adding, or removing some residues, starting from the same NMR model. The termini of the peptides were capped with acetyl and amide groups. These modifications in the peptide molecules were performed using USCF Chimera.^97^ Amino acid substitutions were performed by selecting the most probable rotamer from the backbone-dependent Dunbrack library,^98^ considering steric hindrances with the nearest neighbor atoms. In our simulations, the C-SH2 domain comprised residues from 109 to 217 from the SHP2 wild-type sequence. H-bonds in crystallographic structures were analyzed by using UCSF Chimera. For MD simulations and NMR structure, the persistence values were obtained using VMD^99^ with cutoff criteria of 4 Å for donor–acceptor distance and 20° for donor-hydrogen-acceptor angle.

Typically, in MD simulations, the protonation states of ionizable groups of a protein or peptide are set at the beginning of the simulation and kept constant for the whole trajectory. This approximation can be particularly delicate in the case of side chains whose p*K*_a_ values are close to physiological pH, such as those of H residues. To determine the protonation state of the H side chains present in the C-SH2 domain, the p*K*_a_ value for the second protonation of the imidazole ring was estimated *in silico* with two different methods (PROPKA3.1 ^100,101^ and H++^102^) that account for the effects of the surrounding molecular environment (Table 1). Calculations were performed in the absence and in the presence of a bound phosphopeptide for all available experimental structures of C-SH2/peptide complexes. In all cases, the p*K*_a_ values were significantly lower than the physiological pH of 7.4, suggesting that the neutral state of the side chain is predominantly populated. The only exception was represented by H143. For this residue, only when analyzed using the H++ method and in the presence of ligand, a p*K*_a_ value of 7.6 ± 0.7 was predicted, indicating that an equilibrium between the cationic and neutral state of the side chain might be present. However, in this specific case, there was a discrepancy between the two in silico methods. In addition, H143 is located in close proximity to the pY residue of the peptide ligands (which is common to all sequences), and therefore, its protonation state should not significantly impact any difference observed in the behaviors of the different simulated phosphopeptides. For these reasons, all simulations were carried out with the six H residues in their neutral state.

All MD simulations were performed with the GROMACS 2020.6 software package,^103^ using the AMBER99SB-ILDN force field^104^ augmented with the parm99 data set for pY.^105^ Each protein molecule was put at the center of an octahedral box, large enough to have a distance between the protein and box higher than 1 nm. The protein was solvated with explicit TIP3P^106^ water molecules. The system charge was neutralized with sodium and chloride ions, considering 0.15 M as the saline concentration. Long-range electrostatic interactions were calculated with the particle-mesh Ewald (PME) approach.^107^ A cutoff of 1.5 nm was applied to the direct-space Coulomb and Lennard-Jones interactions. The pressure was set to 1 bar using the weak coupling barostat.^108^ The solvent was relaxed by an energy minimization using the steepest descent algorithm, while restraining the protein and peptide atomic positions. The system was then minimized and slowly equilibrated to the temperature of 300 K using the velocity-rescaling method,^109^ without restraints. The temperature was slowly increased from 50 to 100K with a rate of 1 K/ps and from 100 to 300K with a rate of 0.5 K/ps. A final thermalization at 300K was performed for an additional 500 ps. Finally, a production run of 500 ns was performed for each peptide/domain complex. All of the simulation steps were performed with constraints on covalent bonds and a time step of 2 fs. Each simulation was performed in triplicate; the first 100 ns were excluded from analysis to avoid artifacts due to incomplete conformational rearrangements, and the last 400 ns of each replica were merged to create a unique simulation of 1.2 μs. Analysis of structural properties was performed using GROMACS 2020 analysis tools. Molecular graphics were prepared with UCSF ChimeraX, developed by the Resource for Biocomputing, Visualization, and Informatics at the University of California, San Francisco, with support from the NIH R01-GM129325. For X-ray structures, the RMSF values reported in Figure 4 were obtained starting from the experimentally determined B-factors, through: B=83π2(RMSF)2 .

## Supplementary Material



## Data Availability

All the software tools employed in this study are freely available online. X-ray and NMR structures used in this work were taken from the Protein Data Bank (https://www.rcsb.org/). Protonation state calculations were performed using PROPKA3.1 (https://open.playmolecule.org/tools/proteinprepare) and H++ (version 4.0, http://newbiophysics.cs.vt.edu/H++/). The initial atomic coordinates for the simulations were obtained from the NMR structure with PDB ID 6R5G (https://www.rcsb.org/structure/6R5G) and subsequently modified using UCSF Chimera (version 1.16; https://www.cgl.ucsf.edu/chimera/), as detailed in the Methods section. All molecular dynamics simulations were performed using GROMACS 2020.6 (https://www.gromacs.org/). The force field, input and output .gro files from the simulations, the .mdp file used for the production run, and topology files are available at the Zenodo repository linked here https://zenodo.org/records/15926271. Molecular graphics were prepared using UCSF ChimeraX (version 1.5, https://www.cgl.ucsf.edu/chimerax/). Data plots were generated using the Python library Matplotlib (https://matplotlib.org/). MD trajectories and simulation results were analyzed using GROMACS 2020 built-in analysis tools and Visual Molecular Dynamics (VMD, version 1.9.4; https://www.ks.uiuc.edu/Research/vmd/).

## Notes

Ramachandran plots of the bound peptide residues (Figure S1); ion-pair interactions between peptide residues at position −1 and the C-SH2 domain (Figure S2); ion-pair interactions between peptide residues at position −3 and the C-SH2 domain (Figure S3); ion-pair interactions between peptide residues at position +4 and the C-SH2 domain (Figure S4) (PDF)

## ◆

The authors dedicate this article to the memory of L.S., who passed away on August 24, 2025, and whose inspiration made it possible.
