# Supplementary material for: Decoding C‑SH2 Domain/Peptide Interactions in SH2 Domain-Containing Tyrosine Phosphatase 2: A Molecular Framework for Rational Inhibitor Design
Source: ACS Omega. 2026 Jan 15;11(4):5669–84. doi: 10.1021/acsomega.5c09452 (PMC12878756; doi:10.1021/acsomega.5c09452)
Supplement: Supplementary file 1 [file ao5c09452_si_001.pdf]

Supporting Information

Decoding C-SH2 Domain/Peptide Interactions in SHP2: A Molecular Framework for Rational Inhibitor Design.

Chiara Innamorati,[a] *Layla Bruno,*[a] *Paolo Calligari,*[a,]**Gianfranco Bocchinfuso,*[a,]* *Lorenzo Stella.*[a,♦]

[a] Department of Chemical Sciences and Technologies, University of Rome Tor Vergata, 00133, Rome, Italy

* Prof. Gianfranco Bocchinfuso. Department of Chemical Science and Technologies, Tor Vergata University of Rome, Via della ricerca scientifica, 1, 00133, Rome, Italy.

gianfranco.bocchinfuso@uniroma2.it

* Dr. Paolo Calligari. Department of Chemical Science and Technologies, Tor Vergata University of Rome, Via della ricerca scientifica, 1, 00133, Rome, Italy.

paolo.calligari@uniroma2.it

♦ L.S. Deceased on August 24, 2025

Content:

Figure S1: Backbone conformation of the bound peptide residues

Pag. 3

Figure S2: Ion pair interactions between peptide residues at position -1 and the C-SH2 domain.

Pag. 5

Figure S3: Ion pair interactions between peptide residues at position -3 and the C-SH2 domain

Pag. 6

Figure S4: Ion pair interactions between peptide residues at position +4 and the C-SH2 domain

Pag. 7

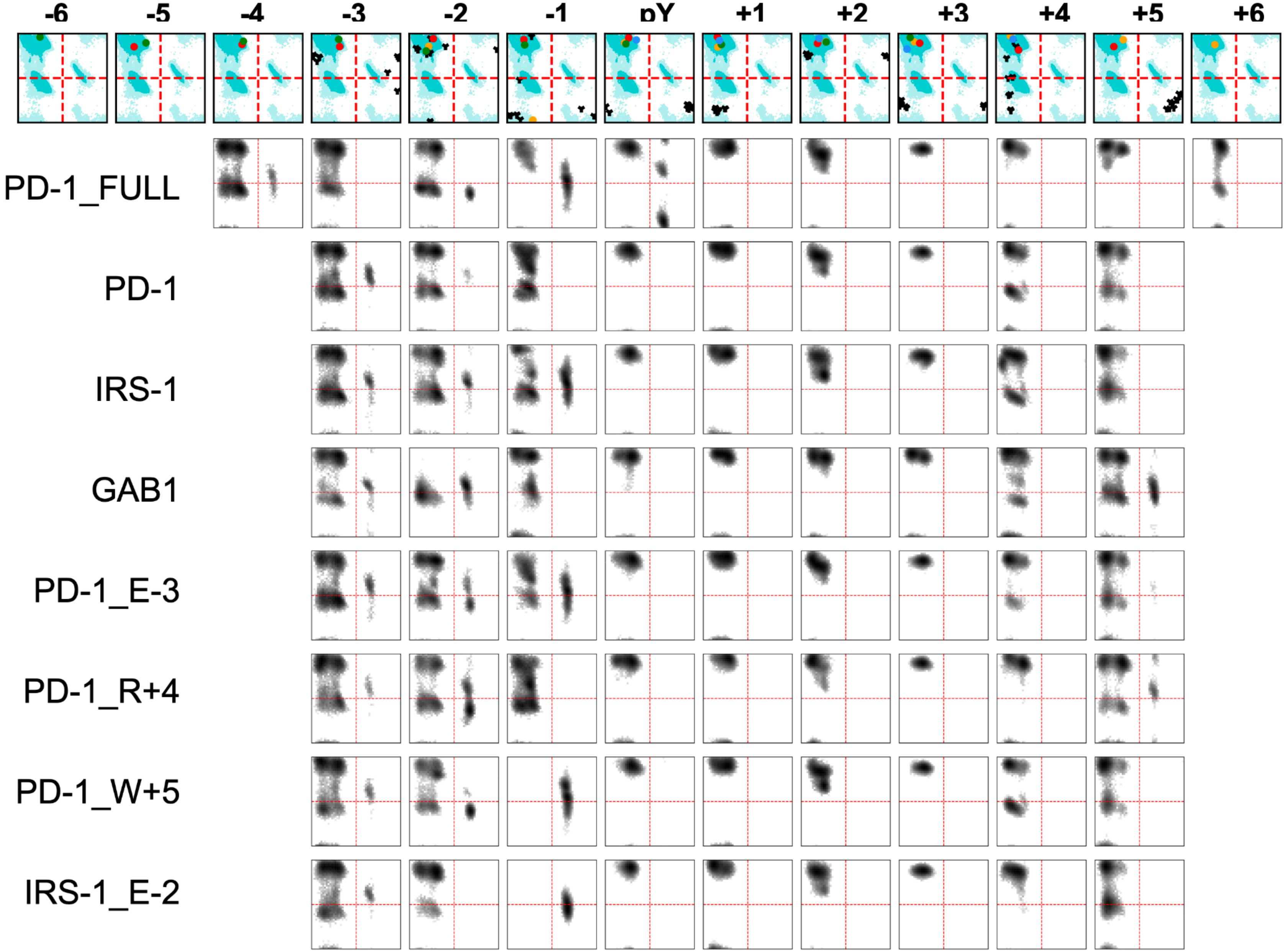

-6
-5
-4
-3
-2
-1
pY
+1
+2
+3
+4
+5
+6
PD-1_FULL
PD-1
IRS-1
GAB1
PD-1_E-3
PD-1_R+4
PD-1_W+5
IRS-1_E-2

Figure S1. Backbone conformation of the bound peptide residues. The Ramachandran plots of the peptide residues in the PDB X-ray and in the NMR structures (first line) and from the MD simulations (below) are shown. Crystallographic structures are reported with the following color code: Eck: orange; 5X94: red; 5DF6: green; 5X7B: blue. NMR models in 6R5G, and simulated conformations are reported in black. In the background of the plots in the first line, the 90 and 99% of allowed values of the Ramachandran plot are highlighted in light and dark cyan, respectively.[94] Angles ϕ and ψ are reported on the x and y axes, respectively, with values from -180° to 180°. The background in the graphs in the first line has been adapted from Ref. 94 in the main text.

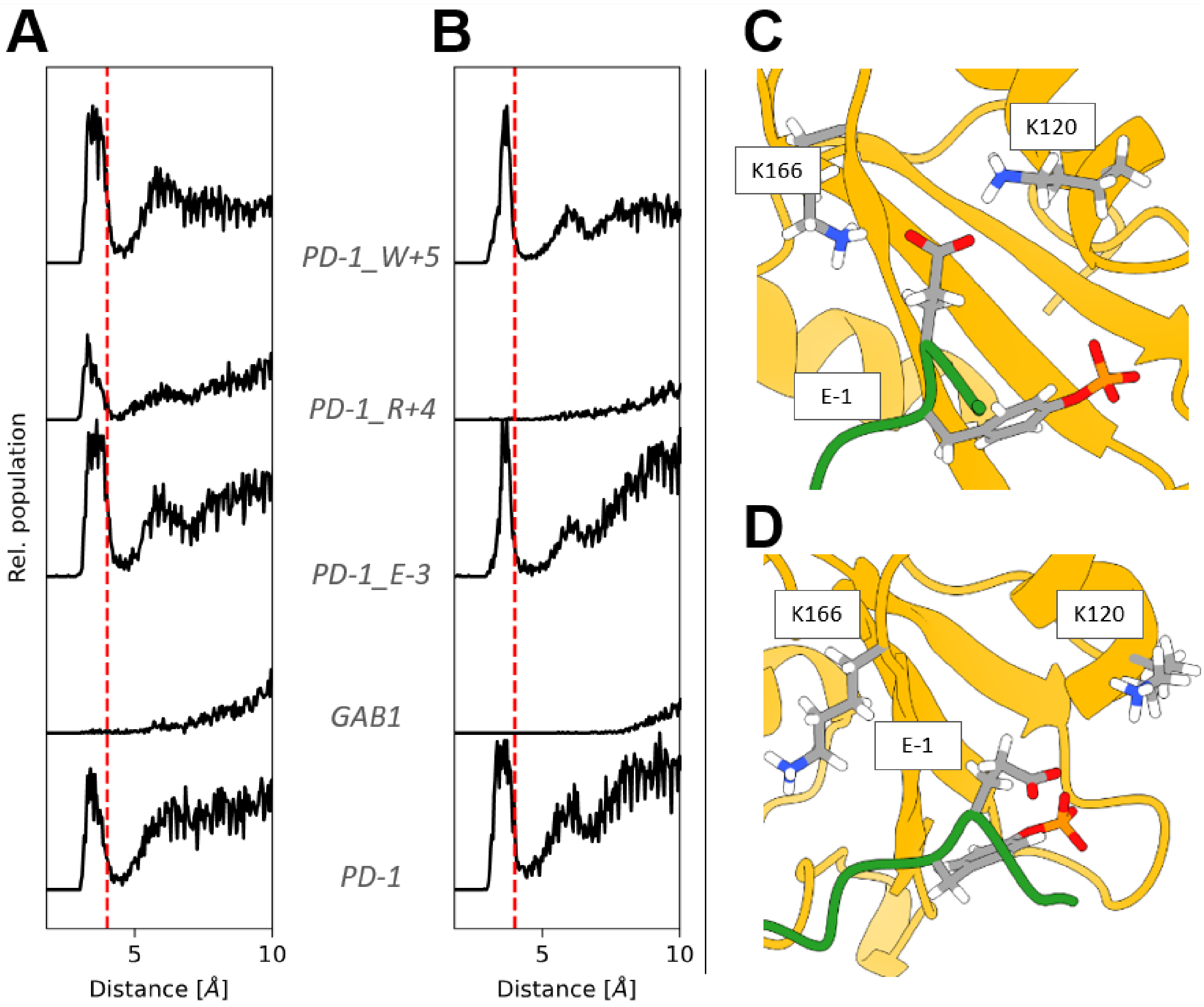

Figure S2. Ion pair interactions between peptide residues at position -1 and the C-SH2 domain. Distribution of distances populated in the MD trajectories between the charged group of anionic residues in -1 and K120 (A) or K166 (B). Distances of less than 4 Å (vertical red dashed lines) are indicative of a stable salt bridge. Structural representations of residues involved in the interactions with focus on K120, K166 and E-1 for the PD-1 (C) and PD-1_R+4 (D) simulations.

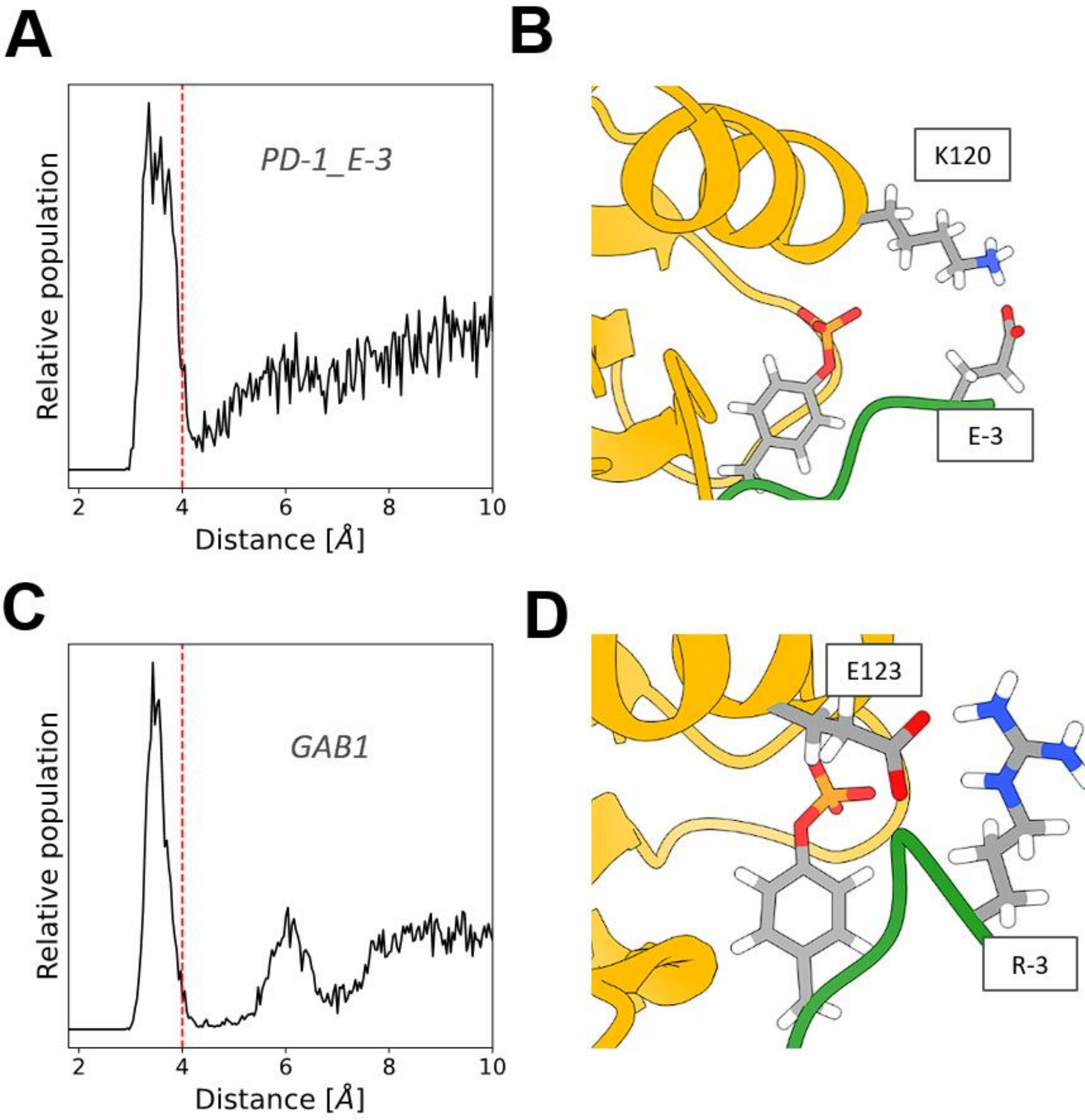

Figure S3. Ion pair interactions between peptide residues at position -3 and the C-SH2 domain. Distribution of charged group distances populated in the MD trajectories for the anionic E-3 and K120 in PD-1_E-3 (A), and for the cationic R-3 and E123 in GAB1 (C). Distances of less than 4 Å (vertical red dashed lines) are indicative of a stable salt bridge. Structural representations of residues involved in the interactions, focus on: K120 and E-1 in the PD-1_E-3 simulation (B), E123 and R-3 in the GAB1 simulation (D).

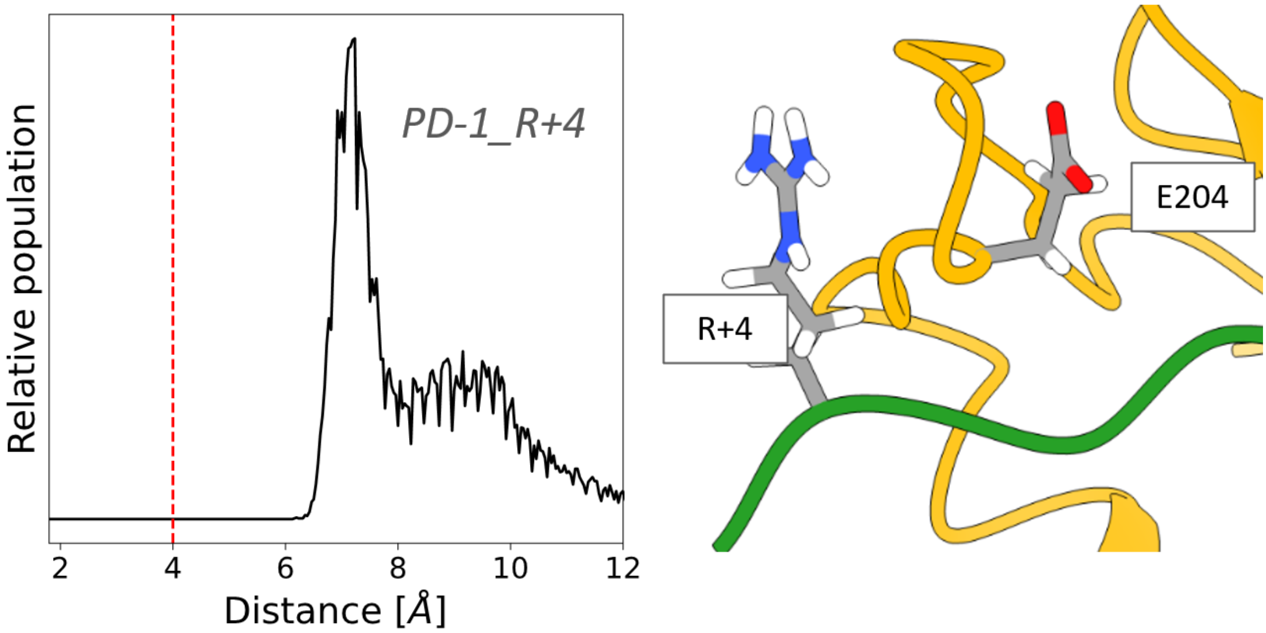

Figure S4. Ion pair interaction between peptide residue at position +4 and the C-SH2 domain-. Distribution of charged group distances populated in the MD trajectories for R+4 and E204 in PD-1_R+4 (left). Distances of less than 4 Å (vertical red dashed lines) are indicative of a stable salt bridge. Structural representations of residues involved in the interaction are shown with focus on E204 and R+4 in the PD-1_R+4 simulation (right).
